# 712 Added Effect, Injuries, And Complications Of Blast Injuries Among Burn Patients On Resuscitation And Outcomes

**DOI:** 10.1093/jbcr/irad045.188

**Published:** 2023-05-15

**Authors:** Anthony Bruccoliere, Bridget Boeger, Alan Pang, John Griswold

**Affiliations:** Texas Tech Health Science Center School of Medicine, Lubbock, Texas; Texas Tech Health Science Center, Lubbock, Texas; Texas Tech University Health Sciences Center, Lubbock, Texas; Texas Tech University Health Sciences Center, Lubbock, Texas; Texas Tech Health Science Center School of Medicine, Lubbock, Texas; Texas Tech Health Science Center, Lubbock, Texas; Texas Tech University Health Sciences Center, Lubbock, Texas; Texas Tech University Health Sciences Center, Lubbock, Texas; Texas Tech Health Science Center School of Medicine, Lubbock, Texas; Texas Tech Health Science Center, Lubbock, Texas; Texas Tech University Health Sciences Center, Lubbock, Texas; Texas Tech University Health Sciences Center, Lubbock, Texas; Texas Tech Health Science Center School of Medicine, Lubbock, Texas; Texas Tech Health Science Center, Lubbock, Texas; Texas Tech University Health Sciences Center, Lubbock, Texas; Texas Tech University Health Sciences Center, Lubbock, Texas

## Abstract

**Introduction:**

Major burn injuries present as complex cases due to the hemodynamic and cardiodynamic changes that occur following the injury which can lead to the development of sepsis, multiple organ failure, and death[1]. Complexity of these cases increase furthermore with the inclusion of explosion induced injuries. Concussive explosive forces can be particularly devastating for air containing organs like the lung, bowel and middle ear [8]. In explosion-related cases, “the mortality correlated significantly with higher burned total body surface area (TBSA), higher abbreviated burn severity index (ABSI) score, accompanying inhalation injuries, and lung contusions” [2]. Cardiac blast traumas may occasionally result directly from blast energy but are more often presented as indirect coronary emboli [4]. Explosive energies have been observed to induce bradycardia, hypotension, and apnea [5]. Although more common in underwater blasts, small bowel perforations can result directly from an explosion or indirectly up to 2 weeks following the explosion [4]. A longer ICU length of stay is also associated with patients suffering from an explosion event [7].

**Methods:**

Through a hypothesis-driven retrospective study, we isolated a group of 68 burn patients, ages 18-85, from 2010-2017 that fit our criteria. Inclusion to the test group required the presence of an injury related to an explosion that resulted in a TBSA% greater than 10%. We then collected data and compared that to the outcomes of patients who had burn injuries not caused by an explosion. The control group had statistically similar age, TBSA percentage, and comorbidities. We compared major outcomes such as length of stay, length of time on a ventilator, and mortality rates.

**Results:**

When controlled for age, TBSA %, presence of inhalation injuries and comorbidities, the explosion group had significantly higher mortality rates (p< 0.001). The length of stay and length of intubation were not significantly different for the explosion group. The incidence of sepsis in the explosion group was nearly significant with a p-value of 0.051.

**Conclusions:**

After analyzing the data, we conclude that patients experiencing an explosion experience higher mortality rates than burn patients of similar age, TBSA%, and comorbidities. The shockwave present in explosion cases likely causes additional injuries to patients that decrease survivability. There was indication that sepsis or infection events may be more likely in patients experiencing an explosion, but results nearly missed the standard of significance. Due to the relatively small sample size of 68 patients, some of the data might be limited in its generalizability. A larger study could provide more insight into the outcomes of patients experiencing explosions.

**Applicability of Research to Practice:**

The effect of explosions on mortality provides an opportunity for quality improvement research and development of improved methods for treating explosion involved burn injuries.

